# Bullous Pemphigoid Triggered by Thermal Burn Under Medication With a Dipeptidyl Peptidase-IV Inhibitor: A Case Report and Review of the Literature

**DOI:** 10.3389/fimmu.2018.00542

**Published:** 2018-04-12

**Authors:** Yosuke Mai, Wataru Nishie, Kazumasa Sato, Moeko Hotta, Kentaro Izumi, Kei Ito, Kazuyoshi Hosokawa, Hiroshi Shimizu

**Affiliations:** ^1^Department of Dermatology, Hokkaido University Graduate School of Medicine, Sapporo, Japan; ^2^Department of Dermatology, JR Sapporo Hospital, Sapporo, Japan; ^3^Department of Dermatology, Shinkotoni Skin Care Clinic, Sapporo, Japan

**Keywords:** bullous pemphigoid, dipeptidyl peptidase-IV inhibitor, dipeptidyl peptidase-IV inhibitor-associated bullous pemphigoid, burn, cellulitis, autoimmune disease, autoantibodies, physical factors

## Abstract

Bullous pemphigoid (BP) is a common autoimmune blistering disease in which autoantibodies mainly target the hemidesmosomal component BP180 (also known as type XVII collagen) in basal keratinocytes. Various triggering factors are known to induce BP onset, including radiotherapy, burns, ultraviolet exposure, surgery, and the use of dipeptidyl peptidase-IV inhibitors (DPP4i), which are widely used antihyperglycemic drugs. Here, we present a case of BP triggered by a thermal burn under medication with DPP4i. A 60-year-old man with type II diabetes had been treated with the DPP4i linagliptin for 1 year. After the right forearm experienced a thermal burn, blisters developed around the burned area and gradually spread over the whole body with the production of autoantibodies targeting the non-NC16A domain of BP180. The diagnosis of BP was confirmed by immunohistopathological examination. Upon withdrawal of linagliptin and treatment with topical steroid and minocycline, complete remission was achieved after 4 months. Previously, 13 cases of BP that developed after thermal burns have been reported, and our case shared some of the clinical features of these thermal burn-induced BP cases. Interestingly, the present case also showed the typical clinical, histopathological, and immunological features of the non-inflammatory type of DPP4i-associated BP (DPP4i-BP). Although the pathogenesis of BP remains uncertain, the present case suggests that DPP4i may trigger the onset of BP similarly to a thermal burn. In addition, the clinical and histopathological features of DPP4i-BP may be distinct from other types of BP.

## Case Presentation

**Figure 1:**
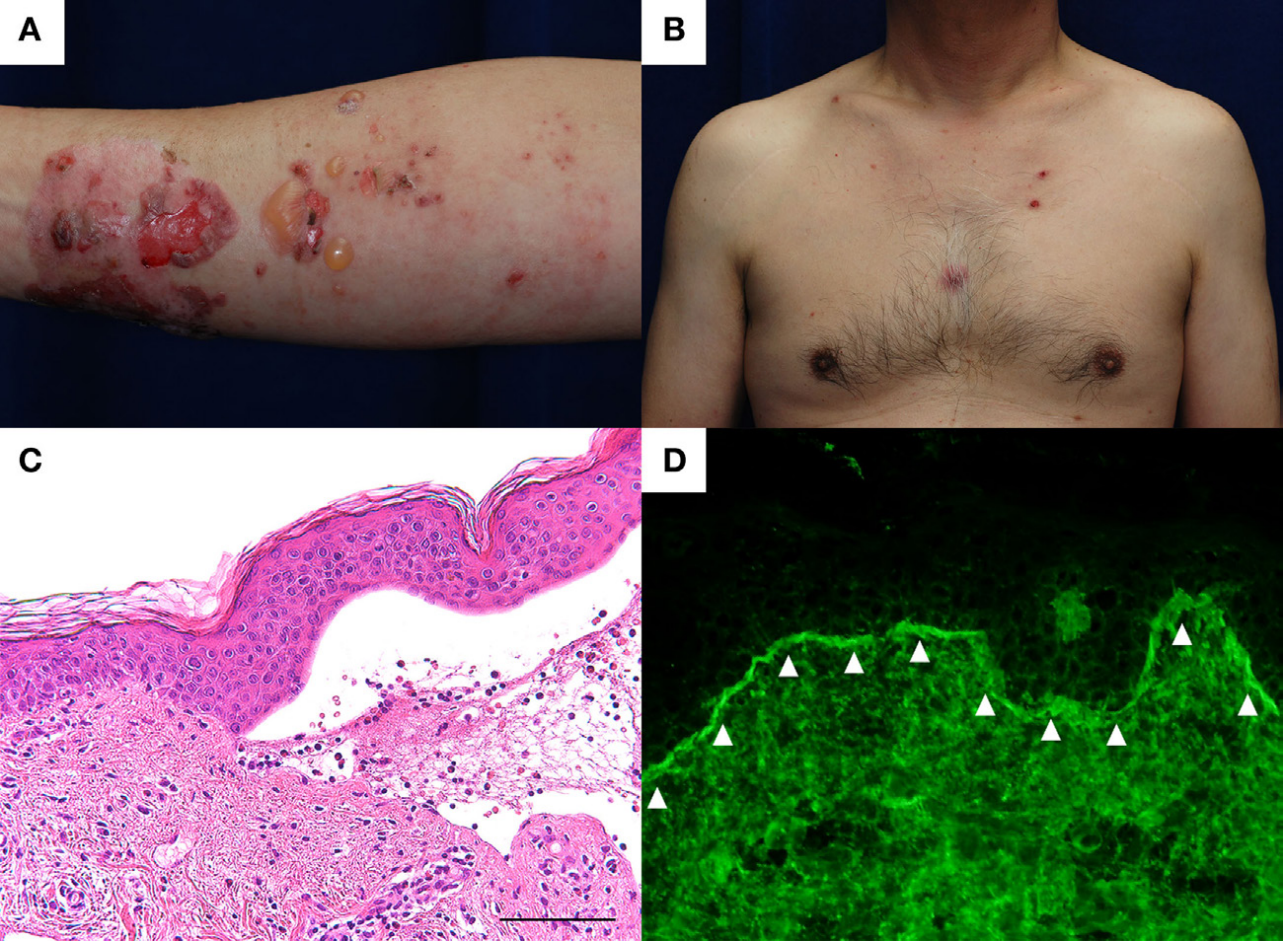
**(A)** Tense blisters and erosions of less than 2 cm in diameter developed over the thermal burn scar on the right forearm. Circumscribed erythematous lesions were also found. **(B)** Small erosions of less than 5 mm in diameter were found on the trunk without erythema. **(C)** Histopathological examination of the blister shows sub-epidermal blistering with some eosinophilic infiltration in the dermis. Scale bar: 100 µm. **(D)** Direct immunofluorescence shows linear IgG autoantibody deposits at the basement membrane zone (arrowheads).

**Figure 2:**
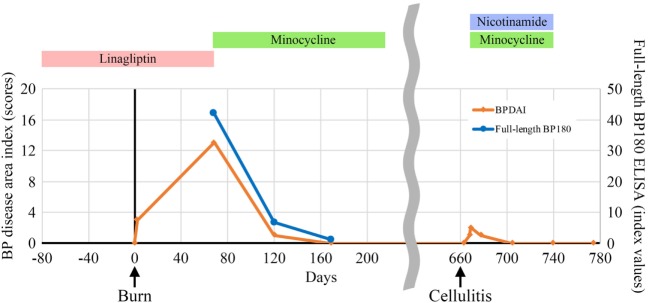
Indices of the bullous pemphigoid (BP) disease area index scores (2) and enzyme-linked immunosorbent assay (ELISA) using full-length recombinant BP180 during the clinical course.

A 60-year-old man with type II diabetes was referred to our department due to blisters and erosions over the whole body. He had been treated with linagliptin, a dipeptidyl peptidase-IV inhibitor (DPP4i), for 1 year. Two months before the referral, he had suffered a deep, 7-cm-long dermal burn on the right forearm from a kitchen accident. Within 2 days after the burn, blisters had appeared on the right forearm, which were treated with a topical antibiotic ointment. However, multiple blisters and erosions gradually developed over the body over the course of 2 months. Physical examination revealed tense blisters and erosions of 5 mm to 2 cm in diameter with circumscribed erythematous lesions predominantly on the right forearm (Figure 1A). Blisters and erosions less than 5 mm in diameter without erythema were found on the face, the trunk, and the left leg (Figure 1B). Although BP180 NC16A chemiluminescent enzyme immunoassay (CLEIA) was negative (5.9 U/mL; normal, <9.0 U/mL), histopathological examination of the blister showed sub-epidermal blister formation with some eosinophilic infiltration in the dermis (Figure 1C). Direct immunofluorescence showed linear deposition of IgG autoantibodies along the dermal–epidermal junction (Figure 1D), and 1 M NaCl-split skin indirect immunofluorescence revealed circulating IgG autoantibodies reacting with the epidermal side of the artificial blisters (not shown). Notably, enzyme-linked immunosorbent assay (ELISA) using full-length recombinant BP180 was positive (index value, 41.8; normal, <4.64) (1). Based on these findings, the diagnosis of bullous pemphigoid (BP) was made. Linagliptin was withdrawn 2 days after the referral, and treatment with a topical steroid and minocycline at 100 mg/day was started. His skin lesions gradually improved, and ELISA with full-length recombinant BP180 became negative (index value, 1.03). Complete remission off therapy was achieved 4 months later (Figure 2). Although no DPP4i was re-administered, blisters appeared on the left forearm 16 months later, when he developed cellulitis on the right leg. The histopathology of the blister showed sub-epidermal blistering with scant eosinophilic infiltration in the dermis (not shown), which was consistent with BP. BP180 NC16A CLEIA was still negative (<3 U/mL). Topical steroid, minocycline at 200 mg/day, and nicotinamide at 1,500 mg/day were initiated, and the lesions completely resolved 2 months later. At 2 months after the cessation of treatment, no recurrence was observed (Figure 2).

## Introduction

Bullous pemphigoid is a common autoimmune blistering disease in which autoantibodies target the hemidesmosomal components BP180 (also known as type XVII collagen) and/or BP230 at the dermal–epidermal junction (3). Clinically, tense blister formation associated with itchy urticarial erythema is typically observed in BP patients, and sub-epidermal blister formation with eosinophilic infiltration is histopathologically observed (3). Although the etiology of BP remains unclear, various factors, including radiotherapy, burns, ultraviolet (UV) exposure, trauma, surgical procedures, topical medications, and infections are known to trigger the onset of the disease (4, 5). Recently, emerging evidence reports that the use of DPP4i, which are widely used to treat patients with diabetes mellitus, increases the risk of BP onset (6–9).

## Discussion

Herein, we report a case of BP in which a thermal burn triggered the onset of the disease. As shown in Table 1, various BP-inducing physical factors, including radiation (5, 10–18), UV radiation (5, 19–30), surgical wounds (5, 31–45), ostomy (31, 46–51), burns (5, 52–62), skin grafts (59, 63–67), and other trauma (5, 68–72) have been reported in the English literature. Regarding thermal burns, 13 cases have been reported (Table 2) (5, 52–61). Notably, in contrast to the present case, none of those 13 cases relapsed (5, 52–61) suggesting that the present case had a unique clinical course.

**Table 1:** List of physical triggering factors for bullous pemphigoid.

Triggering factor	No. of cases
Radiation	38
UV	37
PUVA	13
UVB	5
Goeckerman	4
PUVA and UVB	3
UVA	3
Sunlight	2
PUVA and sunlight	1
PUVASOL	1
Ingram	1
Unknown wavelength	4
Surgical wound	22
Ostomy	19
Colostomy	10
Gastrostomy	5
Ileostomy	2
Urostomy	2
Burn	14
Thermal burn	13
Chemical burn	1
Skin graft	6
Other	11
Scratching	3
Injury	2
Dye injection	1
Mechanical trauma	1
Insect bite	1
Revascularization	1
Photodynamic therapy	1
Hernia	1

*UV, ultraviolet; PUVA, psoralen-ultraviolet A; UVB, ultraviolet B; UVA, ultraviolet A; PUVASOL, psoralen-ultraviolet A and sunlight exposure*.

**Table 2:** Overview of thermal burn-induced bullous pemphigoid (BP) cases.

No.	Age	Sex	BP180	BP230	Diagnosis	Eruption	Delay after burn	Relapse	Reference
1	80	M			HE, DIF, IIF	Localized	2 weeks	Not mentioned	Jevtic and Grigoris (52)
2	69	M			HE	Generalized	3 weeks	Not mentioned	Quartey-Papafio and Hudson (53)
3	75	F			HE	Generalized	10 weeks	No	Balato et al. (54)
4	79	M			HE. DIF, ssIIF	Localized	1 month	No	Vassileva et al. (55)
5	49	F			HE, DIF	Localized	5 weeks	Not mentioned	Wagner et al. (56)
6	67	M			HE, DIF	Generalized	8 months	No	Wagner et al. (56)
7	51	M	NC16A	(−)	HE, DIF, ssIIF	Generalized	6 weeks	No	Xu et al. (57)
8	68	F			HE, DIF, ssIIF	Generalized	A few days	No	Korfitis et al. (38)
9	74	M			HE, DIF	Generalized	2 weeks	Not mentioned	Bachmeyer et al. (58)
10	85	F	NC16A		HE, DIF	Localized	20 months	No	Neri et al. (60)
11	73	F			HE, DIF	Generalized	6 weeks	Not mentioned	Damevska et al. (60)
12	89	F	(−)	(+)	HE, DIF, ssIIF	Localized	69 years	No	Morita et al. (61)
13	76	M	NC16A	(+)	HE, DIF	Generalized	A few hours	Not mentioned	Dǎnescu et al. (5)
Our case	60	M	Non-NC16A		HE, DIF, ssIIF	Generalized	2 days	Yes	

*M, male; F, female; HE, hematoxylin and eosin stain; DIF, direct immunofluorescence; IIF, indirect immunofluorescence; ssIIF, 1 M NaCl-split skin indirect immunofluorescence*.

Interestingly, linagliptin, which is a DPP4i, was adminis-tered to our case until the onset of BP. DPP4i-associated BP (DPP4i-BP) is a unique, recently reported subtype of BP that develops with the administration of DPP4i medications such as vildagliptin, sitagliptin, and linagliptin (6–9). DPP4i-BP tends to show scant erythema and low levels of autoantibodies targeting the NC16A domain of BP180 or the absence of such autoantibodies (1, 73). In our case, BP developed after 1 year of DPP4i administration, in which erythema was scantly observed and autoantibodies specifically targeted the non-NC16A domain of BP180. In addition, complete remission was achieved after the with-drawal of DPP4i and the initiation of treatment with topical steroid and systemic minocycline. Histopathologically, eosinophilic infiltration was not evident. These characteristics closely resembled those of the non-inflammatory type of DPP4i-BP (1, 73); therefore, DPP4i use may also be involved in the development of the disease. It should be noted that a recent study reported that 18% of DPP4i-BP cases relapsed even after the withdrawal of DPP4i (7). Although the immunohistopathological results were uncertain, the present case developed BP-like blisters when he suffered from cellulitis 16 months after complete remission, indicating that DPP4i was also associated with the BP onset in the present case.

The pathogenic mechanisms of the physical trigger for BP onset remain elusive. The physical factors cause tissue destruction that activates the inflammatory process, which may result in the observed auto-reactivity to basement membrane proteins, including BP180 (5). Alternatively, basement membrane proteins may be altered as a result of physical factors (74) resulting in immunogenicity with increased affinity to certain human leukocyte antigen (HLA) alleles (75). Notably, 86% of cases of the non-inflammatory type of DPP4i-BP carried HLA-DQB1*03:01 (73). Although the HLA allele was not examined, it is likely that the present case had a genetic propensity to the breakdown of self-tolerance to BP180.

## Concluding Remarks

In conclusion, we reported a case of BP induced by a thermal burn. Interestingly, the patient received DPP4i and showed the typical clinical, histopathological, and immunological characteristics of the non-inflammatory type of DPP4i-BP. Although the pathogenesis of thermal burn-induced BP and DPP4i-BP remains uncertain, BP may occur due to various triggering factors.

## Ethics Statement

This report on a single patient complies with the Declaration of Helsinki. The patient gave written informed consent for the publication of this report.

## Author Contributions

YM, WN, and KIzumi drafted the paper. KS, MH, KIto, and KH were involved in treating the patient and collecting the clinical data. HS supervised the writing of the manuscript.

## Conflict of Interest Statement

The authors declare that the research was conducted in the absence of any commercial or financial relationships that could be construed as potential conflicts of interest.

## Abbreviations

BP, bullous pemphigoid; DPP4i, dipeptidyl peptidase-IV inhibitor; DPP4i-BP, dipeptidyl peptidase-IV inhibitor-associated bullous pemphigoid; CLEIA, chemiluminescent enzyme immunoassay; ELISA, enzyme-linked immunosorbent assay; HLA, human leukocyte antigen.
